# Dosing Therapeutic Radiopharmaceuticals in Obese Patients

**DOI:** 10.3390/ijms23020818

**Published:** 2022-01-13

**Authors:** Merel van Nuland, Tessa F. Ververs, Marnix G. E. H. Lam

**Affiliations:** 1 Department of Clinical Pharmacy, University Medical Center Utrecht, Heidelberglaan 100, 3584 CX Utrecht, The Netherlands; vnuland.m@gmail.com (M.v.N.); F.F.T.Ververs@umcutrecht.nl (T.F.V.); 2 Department of Radiology and Nuclear Medicine, University Medical Center Utrecht, Heidelberglaan 100, 3584 CX Utrecht, The Netherlands

**Keywords:** obesity, radiopharmaceutical, pharmacokinetics

## Abstract

The prevalence of obesity has increased dramatically in the Western population. Obesity is known to influence not only the proportion of adipose tissue but also physiological processes that could alter drug pharmacokinetics. Yet, there are no specific dosing recommendations for radiopharmaceuticals in this patient population. This could potentially lead to underdosing and thus suboptimal treatment in obese patients, while it could also lead to drug toxicity due to high levels of radioactivity. In this review, relevant literature is summarized on radiopharmaceutical dosing and pharmacokinetic properties, and we aimed to translate these data into practical guidelines for dosing of radiopharmaceuticals in obese patients. For radium-223, dosing in obese patients is well established. Furthermore, for samarium-153-ethylenediaminetetramethylene (EDTMP), dose-escalation studies show that the maximum tolerated dose will probably not be reached in obese patients when dosing on MBq/kg. On the other hand, there is insufficient evidence to support dose recommendations in obese patients for rhenium-168-hydroxyethylidene diphosphonate (HEDP), sodium iodide-131, iodide 131-metaiodobenzylguanidine (MIBG), lutetium-177-dotatate, and lutetium-177-prostate-specific membrane antigen (PSMA). From a pharmacokinetic perspective, fixed dosing may be appropriate for these drugs. More research into obese patient populations is needed, especially in the light of increasing prevalence of obesity worldwide.

## 1. Introduction

Over recent decades, the prevalence of obesity (body mass index, BMI ≥ 30 kg/m^2^) has increased dramatically in the Western population [1]. According to the World Health Organization (WHO), the global prevalence of obesity is over 650 million adults (~13% of adults) [2]. If the trend continues, about 18–21% of adults are estimated to be obese by 2025 [1].

Obesity is known to influence the proportion of adipose tissue, which may increase the volume of distribution for lipophilic drugs. Furthermore, it also influences physiological processes such as gastric emptying and cardiac output, the number of plasma proteins, and renal blood flow [3]. These physiological variations could alter drug pharmacokinetics as well as pharmacodynamics. Therefore dose adaptions may be required for patients with obesity, especially patients with morbid obesity (BMI ≥ 40 kg/m^2^) [4].

Despite the high global prevalence of obesity, specific dosing strategies in this patient population are limited [4]. This also holds true for dosing of radiopharmaceuticals that are widely used for diagnostic imaging and radionuclide therapy. To date, practical dosing guidelines for radiopharmaceuticals in obese patients are not available. For some radiopharmaceuticals, the European Association of Nuclear Medicine (EANM) sets maximum doses, such as for lutetium-177 (^177^Lu)-prostate-specific membrane antigen (PSMA), but does not refer in their guidelines to overweight patients [5]. The impact of obesity on diagnostic nuclear imaging has been evaluated, showing that obesity affects the quality of nuclear images [6]. The authors recommended that the effect may be minimized by special preparations, such as lengthening the acquisition time, to improve imaging outcomes. However, the influence of obesity on the efficacy and safety of therapeutic radionuclides is rarely subject of investigation.

The group of radiopharmaceuticals are composed of radionuclides for therapeutic use, such as radium-223 (^223^Ra), and peptide receptor radionuclide therapy (PRRT), such as (^177^Lu)-dotatate. Bone-seeking radionuclides ^223^Ra and strontium-90 (^89^Sr) are substitutes for calcium and selectively incorporate in the bone matrix with high osteoblastic activity [7,8,9], while sodium iodide-131 (^131^I) is trapped in the thyroid gland [10]. PRRTs, such as samarium-153-ethylenediaminetetramethylene ((^153^Sm)-EDTMP), rhenium-186-hydroxyethylidene diphosphonate ((^186^Re)-HEDP), (^131^I)-metaiodobenzylguanidine (MIBG), (^177^Lu)-dotatate, and (^177^Lu)-prostate-specific membrane antigen (PSMA) are radionuclide-peptide conjugates that selectively bind a target receptor [11,12,13,14,15]. All radiopharmaceuticals cause cell damage by emitting radioactivity in the target tissue. The mechanisms of action and target organs of each radiopharmaceutical are shown in Table 1.

A therapeutic dosing regimen and the used body size descriptor varies with drug characteristics and type of treatment. The most commonly used body size descriptor is total body weight (TBW). In addition, lean body weight (LBW), sometimes called fat-free mass (FFM), may be used to calculate weight-based dosages [4]. LBW can be calculated using the Equations (1) and (2):

(1) $$\mathrm{LBW}\;\left(\mathrm{male},\;\mathrm{kg}\right)=\frac{9.27\times 10^{3}\times \;\mathrm{TBW}\left(\mathrm{kg}\right)}{6.68\times 10^{3}+216\times \;\mathrm{BMI}\left(\frac{\mathrm{kg}}{m^{2}}\right)}$$

(2) $$\mathrm{LBW}\;\left(\mathrm{female},\;\mathrm{kg}\right)=\frac{9.27\times 10^{3}\times \;\mathrm{TBW}\left(\mathrm{kg}\right)}{8.78\times 10^{3}+244\times \;\mathrm{BMI}\left(\frac{\mathrm{kg}}{m^{2}}\right)}$$

The aim of this review was to summarize available data on the impact of obesity on the effect and adverse effects of therapeutic radionuclides, and to discuss different dosing strategies in obese patients in nuclear medicine. Literature of available systemically administered radiopharmaceuticals will be discussed individually and categorized by treatment targets, which translate into practical guidelines for dosing of radiopharmaceuticals in obese patients.

## 2. Literature Search

Pubmed and Embase were searched systematically for literature regarding radiopharmaceutical therapy dosing in obese patients, using the term ‘obesity’ in combination with the different drugs. This search only identified one article in which the impact of BMI on the survival of patients with thyroid cancer was described [16]. Therefore, the search was expanded to gather information from dose-escalation studies of radionuclide therapies. The literature search was performed on 1 October 2021 and results were restricted to English language. Furthermore, Summaries of Product Characteristics (SmPC), FDA clinical pharmacology and biopharmaceutics reviews, and the European Public Assessment Reports (EPAR) were consulted. Additionally, citation snowballing was used to find other relevant studies. Publications were initially screened based on title and abstract. Inclusion was performed manually by full-text assessment of eligibility.

### Level of Evidence

Per drug, literature was classified based on the classification system provided by the Oxford Centre for Evidence-Based Medicine (CEBM) [17]. The following levels were defined:

1. Randomized controlled trial (RCT) in obese patients;
2. Case-control study, retrospective comparative study, and systematic review of level three studies;
3. For weight-based dosing regimen: dose-escalation studies with dose interval exceeding registered dose;

For fixed-dose regimen: obese patients included in clinical trials;

4. Case series;
5. Expert opinion.

## 3. Bone-Targeting Agents

Of the radiopharmaceuticals that are registered for treatment of bone metastases, ^223^Ra) and ^153^Sm)EDTMP are dosed on TBW. For ^89^Sr, calculating the dose on LBW is proposed in heavyweight patients, whereas ^186^Re-HEDP is given in a fixed dose. After intravenous administration, these drugs mimic calcium and selectively accumulate in areas of increased bone turnover that surround metastatic lesions [18]. Bone tissue is part of the LBW. There is debate on the correlation between obesity and bone mineral density (BMD). The majority of studies have shown that being obese may have a protective effect on skeletal health by increasing BMD [19,20]. This may be attributed to increased levels of vitamin D, estrogen, insulin, leptin, and proinflammatory cytokines that stimulate bone growth [19,21]. However, other studies have shown a negative association between obesity and bone mass, including an increased risk of fractures [20,22]. Based on inconclusive literature, it is not possible to estimate the effect of obesity on BMD. However, literature agrees that bone mass does not increase to the same extend as the TBW in obese patients. Theoretically, dosing of radiopharmaceuticals on TBW in obese patients could lead to drug toxicity due to high levels of radioactivity. On the other hand it may also lead to a better effect.

### 3.1. Radium-223

^223^Ra, an alpha-emitting agent, has a significant role in treating symptomatic skeletal metastases from prostate cancer [23]. Clinically, ^223^Ra is given at a dose of 55 kBq/kg every 4 weeks for six doses. The distribution of ^223^Ra is rapid by either uptake in bone tissue or hepatic clearance. Four hours after administration, ~61% of the radioactivity is present in bone, ~49% in the bowel, and ~4% in blood. The majority of ^223^Ra is cleared hepatically, with 76% of the administered radioactivity being excreted within 7 days after administration. These data were obtained from a study with 16 patients in a dose-range of 55–221 kBq/kg. [7].

Registration studies have provided information on safety and efficacy in patients receiving high-dose ^223^Ra. In a phase 1 trial (n = 25), dosages up to 276 kBq/kg were administered [24]. At each dose level (n = 5), ^223^Ra was well tolerated without dose-limiting toxicities being observed after single-dose administration. Furthermore, the effect of body mass index (BMI) and weight on efficacy and safety of ^223^Ra were evaluated in subgroup analyses from the pivotal trial (ALSYMPA trial) [23]. In patients with a BMI ≥ 30 mg/m^2^ median overall survival was higher in 153 patients who received ^223^Ra compared to 78 patients who received placebo (16.1 vs. 12.6 months) with a hazard ratio (HR) of 0.617 (95% confidence interval (CI) 0.431–0.883). Furthermore, data have shown an increased survival in patients with a BMI ≥ 30 mg/m^2^ (n = 153) compared to patients with a BMI < 30 mg/m^2^ (n = 434) (16.1 vs. 14.1 months). Treatment groups were compared to placebo, but no statistical comparison was made between patients with a BMI above and below 30 kg/m^2^, and therefore no *p*-value or HR can be reported. The incidence of adverse events was similar in both treatment groups and comparable to that in the placebo arm [7].

Further subgroup analyses of the ALSYMPA were performed for different weight groups. The original FDA application included a subgroup analysis in three groups: patients with TBW < 80 kg, 80–100 kg, and >100 kg. In patients with a TBW > 100 kg, median overall survival was higher in 65 patients who received ^223^Ra compared to 37 patients who received placebo (21.7 vs. 11.8 months) with a hazard ratio (HR) of 0.344 (95% confidence interval (CI) 0.180–0.658). Furthermore, an increased survival was seen in 153 patients with TBW > 100 kg and 284 patients with TBW 80–100 kg compared to 261 patients with a TBW < 80 kg (21.7 vs. 51.4 vs. 13.2 months). Again, no statistical comparison was made between these groups as the survival data were only compared to placebo [7].

On request of FDA reviewers, an additional subgroup analysis was performed in four weight groups: patients with TBW ≤ 73 kg, 73–82 kg, 82–91 kg, and >91 kg. Survival analysis showed an increased survival for the highest-weight group, and thus the authors concluded that increased body weight is related to better overall survival. The Pharmacology and Biopharmaceutics review provides Kaplan–Meier curves, but no survival-outcome data. Therefore, no time-to-event data were reported. In this analysis, no evident relationship was found between body weight and safety of ^223^Ra treatment. A logistic regression model did not show a correlation between thrombocytopenia and body weight. Based on these data, higher body weight (>100 kg) seems to be related to increased overall survival and higher chance of treatment response, possibly due to the higher level of radioactivity administered to these patients [7].

**Figure 1:**
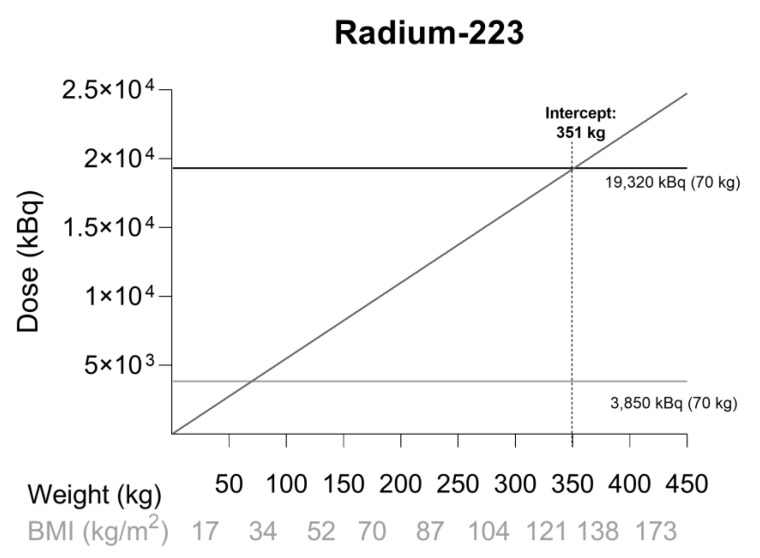
Dose simulation for radium-223 (^223^Ra) at 55 kBq/kg. The gray horizontal line represents the registered dose for a 70 kg patient (55 kBq/kg), while the black horizontal line represents the maximum tolerated dose for a 70 kg patient (276 kBq/kg). The BMI was calculated for a patient with average height (1.70 m). The intercept at 351 kg shows the weight at which the registered dose is equal to the maximum tolerated dose when administered to a 70 kg patient. This corresponds to a patient with a BMI of 121 kg/m^2^.

Given the data from subgroup analyses, we may conclude that the efficacy and safety of ^223^Ra has been sufficiently established in obese patients. Even more, a higher radioactivity dose in patients >100 kg may lead to prolonged overall survival without an increased toxicity profile. Figure 1 shows a graph of weight versus ^223^Ra dose when administered at 55 kBq/kg. The intercept at 351 kg is the weight at which the dose equals the maximum tolerated dose when administered to a 70 kg patient. This corresponds to a patient with a BMI of 121 kg/m^2^ assuming average height (1.70 m). Based on these data it is recommended to calculate ^223^Ra dose on TBW, regardless of BMI.

### 3.2. Samarium-153-EDTMP

^153^Sm emits beta particles of 0.81 MeV (20%), 0.71 MeV (30%), and 0.64 MeV (50%). It is complexed to the bone-seeking phosphate EDTMP. In the clinic, (^153^Sm)-EDTMP is administered at doses of 37 MBq/kg, which can be repeated every 8 weeks [11]. After intravenous administration, (^53^Sm)-EDTMP is rapidly eliminated from plasma, with only 10% of radioactivity left in plasma after 30 min [25]. Pharmacokinetic studies have shown that 50% is recovered in bone tissue. The remainder is excreted rapidly via urine; 30% of (^153^Sm)-EDTMP is recovered in urine within 4 h, and 35% within 12 h [26,27,28].

No studies in obese patients were available, but two dose-escalation studies have been published in which a large dose-range was studied. In the first study, (^153^Sm)-EDTMP was administered to 22 patients in a dose-range of 3.7–37 MBq/kg [25]. A decline in platelet count was observed at doses ≥ 13 MBq/kg, and a decline in white blood cells at doses ≥ 28 MBq/kg. Treatment response occurred in 60% of patients injected with 3.7–13 MBq/kg and in 69% of patients injected with 18.5–37 MBq/kg, although this was not statistically significant (*p* = 0.692). In the second dose-escalation study 52 patients were treated in a dose-range of 37–111 MBq/kg [26,29]. Patients received dosages between 1887 and 11,063 MBq. The highest dose was administered at dose level 93 MBq/kg, which back-calculated to a patient weight of 119 kg. The maximum tolerated dose was set at 93 MBq/kg as two out of four patients in dose level 111 MB/kg had developed grade 3 hematologic toxicity (neutrophil count 500–900/mm^3^). For further toxicity and response evaluation, dose levels 37 MBq/kg and 93 MBq/kg were expended with 16 additional patients. Patients who received 93 MBq/kg (n = 20) showed increased hematologic toxicity compared to those who received 37 MBq/kg (n = 20), with a lower neutrophil count (1000/mm^3^ versus 2100/mm^3^, *p* < 0.001), lower platelet count (65,000/mm^3^ versus 132,000/mm^3^, *p* < 0.001) and lower hemoglobin levels (1.6 g/dL vs. 2.8 g/dL, *p* < 0.01) [29]. Hematologic recovery of neutrophils and platelets occurred naturally in 45 of 52 patients (87%). Although toxicity was more pronounced in the higher-dose level, there was a significant increase in treatment response. Survival of patients receiving 93 MBq/kg was significantly longer compared to patients receiving 37 MBq/kg (9 vs. 6 months, *p* = 0.03). Furthermore, there was a significant improvement in self-report of pain in the 93 MBq/kg dose level versus the 37 MBq/kg dose level (*p* = 0.024), and in opioid use over the study period (*p* = 0.015) [29].

**Figure 2:**
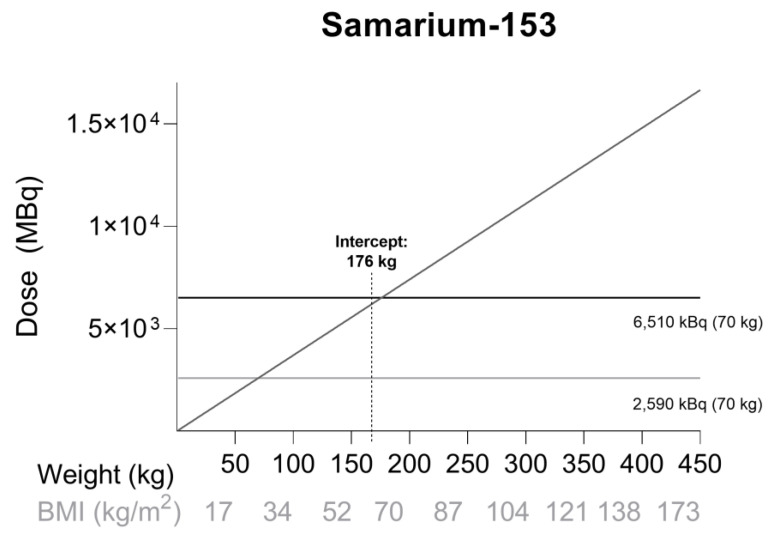
Dose simulation for samarium-153 (^153^Sm) at 37 kBq/kg. The black horizontal line represents the recommended dose for a 70 kg patient (37 kBq/kg), while the gray horizontal line represents the maximum tolerated dose for a 70 kg patient (93 kBq/kg). The BMI was calculated for a patient with average height (1.70 m). The intercept at 176 kg shows the weight at which the registered dose is equal to the maximum tolerated dose when administered to a 70 kg patient. This corresponds to a patient with a BMI of 61 kg/m^2^.

Based on the dose-escalation data, higher dosages are associated with prolonged survival, but also with increased hematologic toxicity. No data has yet been reported on (^153^Sm)-EDTMP treatment in obese patients. Future studies need to explore the impact of obesity on efficacy and safety of (^153^Sm)-EDTMP. In the absence of clinical data, information from dose-escalation studies can be used for dosing recommendations in obese patients. Dose level 93 MBq/kg was considered effective and safe in terms of overall survival and hematologic toxicity [29]. This dose level is three times the registered dose of 37 MBq/kg. Figure 2 shows a weight–dose graph for (^153^Sm)-EDTMP when administered at 37 MBq/kg. The intercept at 176 kg shows the weight at which the registered dose is equal to the maximum tolerated dose when administered to a 70 kg patient. This corresponds to a patient with a BMI of 61 kg/ m^2^ assuming average height (1.70 m). These data suggest that 37 MBq/kg TBW may be used for treatment of obese patients under close monitoring of hematologic toxicity.

### 3.3. Strontium-89

^89^Sr is an alpha-emitting agent which is used for treatment of bone metastases in patients with cancer [8]. The recommended dose of ^89^Sr is fixed at 150 MBq or weight-based at 1.5–2.2 MBq/kg. According to the SmPC, in particularly light- or heavyweight patients, a dose of 2 MBq/kg fat-free body weight is recommended [8]. Following intravenous injection, ^89^Sr rapidly distributes to bone mineral where it emits β-energy with a maximum energy of 1.463 MeV. ^89^Sr is mainly excreted renally (80%) [8,30]. The biological half-life is 14 days [8].

According to the SmPC, ^89^Sr should be dosed on fat-free body weight in heavyweight patients [8]. No further specifications are given on the definition of a heavyweight patient. The basis for this dosing regimen was derived from animal experiments; in rat and rabbit studies, ^89^Sr was predominantly absorbed in bone tissue, while the activity in fat tissue was negligible. If the therapeutic dose is titrated to body mass, than obese patients (e.g., >35% body fat) would receive a high dose relative to the actual distribution of drug [31]. Interestingly, the efficacy and safety of this dosing regimen compared to dose calculation based on TBW has not been studied in human patients. Moreover, defining the ^89^Sr dose on fat-free body weight in heavyweight patients might lead to underdosing in obese patients. For example, a patient with BMI = 30 (height = 1.70 m and weight = 86.7 kg) will have a calculated LBW of 61 kg. Based on the recommended dosing regimen, this patient would receive 122 MBq instead of 150 MBq. As such, dosing on fat-free body weight may only be of additional value in patients with LBW > 75 kg, as the total dose will then exceed the fixed-dosing regimen of 150 MBq as commonly used in clinical practice.

Administered ^89^Sr doses ranged from 0.56 to 6.85 MBq/kg in different clinical studies [32,33,34,35,36]. Robinson et al. were major contributors to clinical studies involving ^89^Sr, and they showed an increased response rate with increasing dose. The authors reported a threshold dose of approximately 1.11 MBq/kg [37]. In their largest study, 20 patients received 1.11 MBq/kg followed by 182 patients who received 1.48 MBq/kg. The overall response rate in terms of pain relief and improved quality of life was 80%. Hematologic toxicity was seen in 80% of patients, with a mean decrease of 15–20% in platelet and white blood cell count [32]. Based on these data, the minimum effective dose was determined to be 1.48 MBq/kg. Laing et al. reported on a dose-escalation study in which 117 patients received ^89^Sr doses of 1.5–3 MBq/kg [36]. They showed no clear benefit from dose increase in terms of cumulative mortality. However, there was a significant increase in the percentage of platelet depression with increasing dose, up to 45% at a dose level of 3 MBq/kg (*p* < 0.02) [36]. Based on a subsequent dose estimation study, a fixed dose of 150 MBq was recommend for therapeutic application of ^89^Sr [36]. The highest MBq/kg dose administered in literature was reported by Kloiber et al. Ten patients received doses ranging from 2.81 to 6.85 MBq/kg [35]. Five out of the ten patients (50%) showed improvement as measured by general condition, level of mobility, and pain analysis. Nonetheless, all patients showed a reversible reduction in platelet count of 24–66%. The authors did not report data related to the administered dose [35].

Taking all studies into account, doses of ~1.5 MBq/kg show high efficacy and tolerable adverse events. Further dose increment to 3 MBq/kg did not improve cumulative mortality, while causing an increase in platelet depression. Based on these data, it should not be recommended to dose ^89^Sr on TBW in obese patients. Although there is no literature available of dosing ^89^Sr in obese patients, both a fixed-dosing scheme and dosing at 2 MBq/kg LBW > 75 kg seems appropriate. This needs yet to be supported by clinical data.

### 3.4. Rhenium-186-HEDP

^186^Re can be used in two radiopharmaceutical formulations, being ^186^Re-HEDP and ^186^Re sulfide colloid. Currently, this drug is no longer available, but is included in this review for completeness. The sulfide colloid is used for local treatment of radiosynoviorthesis by intra-articular injections at fixed doses of 185 or 370 MBq [38], and is outside the scope of this review (because it is not administered systemically). ^186^Re emits beta particles of 1.07 mEv and is complexed to HEDP to form a bone-seeking complex. The recommended dose for (^186^Re)-HEDP is fixed at 1110–1295 MBq [14]. (^186^Re)-HEDP is cleared primarily by urinary excretion [39]. A pharmacokinetic study showed that 70% of the drug is recovered in urine at 24 h after intravenous injection (n = 17) [40].

In a first-in-human dosimetry and biodistribution trial, published in 1989, a mean single intravenous dose of 174 MBq was administered to five patients with skeletal metastases [39]. By linear extrapolation and by applying dosimetry models on data from this diagnostic study, a therapeutic dose of 925–1295 MBq was established, which would deliver an average of 10–140 Gy to metastatic lesions [14,39,41,42]. In follow-up trials with therapeutic (^186^Re)-HEDP, a single intravenous dose of 1221–1258 MBq was administered to patients with bone metastases [14,39,41,42]. The pharmacokinetics of (^186^Re)-HEDP were determined at three different dosages: 1262 MBq (n = 13), 1828 MBq (n = 3), and 2353 MBq (n = 1) [40]. All doses were well tolerated.

The weight of included patients was not reported in these publications. Therefore, recommendations for dosing of (^186^Re)-HEDP in obese patients have to be based solely on pharmacokinetics. (^186^Re)-HEDP accumulates at sites of increased bone turnover that surround metastatic lesions with a volume of distribution in plasma of 1.1 L/kg at steady state [39,40]. Considering these pharmacokinetic properties, a fixed dose may be appropriate for all patients, including obese patients.

## 4. Antithyroid Treatment

Iodide-131 may be used for treatment of benign thyroidal disease (e.g., hyperthyroidism characterized by excess concentration of circulating thyroid hormones), and for treatment of thyroid cancer [43]. Thyroid tissue is part of the LBW and will not increase in obese patients. In general, thyroid function is normal in obese patients [44]. On the other hand, hypothyroidism is linked to weight gain, which causes a positive association between serum thyroid stimulating hormone (TSH) levels and BMI [45]. ^131^I accumulates in the thyroid due to high affinity for the target organ. For radionuclide treatment with high accumulation in target tissue, fixed dosing could be an adequate dosing strategy. However, there is a risk of underdosing for lipophilic drugs with high levels of distribution to adipose tissue. Furthermore, caution should be exercised to avoid toxicity of lung parenchyma and bone marrow due to high radioiodine uptake [43].

### Sodium Iodide-131

^131^I is approved for treatment of hyperthyroidism. It was first administered as a so-called ‘anatomic cocktail’ in 1946 [45]. Different doses are registered for treatment of benign and malignant diseases. For benign and malign application, doses generally range between 0.2 and 0.8 GBq, and 1.85 and 1.11 GBq, respectively [10]. There is no maximum defined dose for treatment of benign hyperthyroidism, while the maximum dose for malign application is 7.4 GBq per cycle without a maximum number of treatment cycles. There is debate on whether the dose should be fixed or individualized based upon the size of the thyroid gland. When an individualized dose is administered, the activity depends on the diagnosis, size of the gland, thyroid uptake and iodine clearance. Generally, radioiodine activities are fixed and based on disease characteristics and patient age [43]. ^131^I decays by beta emission (191.6 KeV) and associated gamma emission (364.5 KeV) [46].

After oral administration, 90% of the drug is absorbed within 60 min after administration. After both oral and intravenous administration, ^131^I distributes to extracellular fluids and is trapped by the thyroid [46]. Approximately 20% of iodide is taken up by the thyroid gland in one pass. Concentrations up to 500-times the plasma concentrates may be achieved in the thyroid gland. Other critical organs are the stomach, plexus, and salivary glands. Furthermore, radioiodine uptake has been described in a variety of tissues, predominantly at metastatic or inflammatory sites [47,48]. Upon uptake in the thyroid, iodide is further oxidized to iodine, which organically binds thyroid tissue [46]. ^131^I is 37–75% excreted renally with a biological half-life of 12 h in plasma, and 6 days in the thyroid gland [43].

Al-Ammar et al. studied the impact of BMI on the survival of patients with thyroid cancer. Of 209 included patients, 156 patients received adjuvant sodium iodide in a dose-range of 1.11–7.4 GBq [16]. Of the total population, 78.1% had a BMI > 26 and were considered overweight. Data analyses were performed with a combined population of patients receiving adjuvant sodium ^131^I and patients with solely thyroidectomy or neck dissection. Results may apply to obese patients receiving ^131^I, assuming equal distribution of obese patients in both groups. The study showed no impact of BMI on treatment outcome, defined as disease-free survival and overall survival [16].

No dose-escalation studies have been described in literature. Following absorption, iodide is primarily distributed within body fluids. In parallel, sodium ^131^I accumulates in thyroid tissue. Based on the distribution profile of ^131^I and its minimal toxicity profile, a fixed dose may be appropriate for treatment of patients with obesity.

## 5. Peptide-Receptor Radiopharmaceuticals

Several radiopharmaceuticals, including (^131^I)-MIBG, (^177^Lu)-dotatate), and (^177^Lu)-PSMA may be used for treatment of different types of cancer. (^131^I)-MIBG is registered for treatment of neuroendocrine tumors, including paragangliomas, pheochromocytomas, and carcinoid tumors in a fixed dose of 3.7–7.4 GBq [13]. (^177^Lu)-dotatate is approved for treatment of gastroenteropancreatic neuroendocrine tumors (GEP-NETs) in a fixed dose of 7.4 GBq per cycle, up to four administrations [12]. Finally, (^177^Lu)-PSMA is a promising treatment of metastatic castration-resistant prostate cancer in a dose of 7.4 GBq per cycle, with a maximum of six cycles [49]. All four drugs target tumor-specific proteins and accumulate selectively in tumor tissue after intravenous administration.

### 5.1. Iodide-131-MIBG

MIBG is structurally similar to norepinephrine and specifically targets neuroendocrine tumors such as neuroblastoma, pheochromocytoma, and carcinoid tumors [13,50]. When labeled to ^131^I, it may be used as a therapeutic agent for patients with these tumor types. Neuroblastoma is a rare cancer that mainly affects children under age five and is the primary indication for (^131^I)-MIBG treatment. For all three indications, single-administered activities vary from 3.7–11.2 GBq [51], although the registered dose is 3.7–7.4 GBq [13]. For neuroblastoma specifically, children are given two administrations with a dose interval of 4 weeks, with a total dosimetry-based bone marrow dose of 4 Gy. ^131^I is a beta emitter (191.6 KeV) and consequently produces gamma radiation (364.5 KeV) [13]. Free ^131^I rapidly accumulates in the thyroid gland, therefore, prophylactic thyroid blockage with stable iodine is part of standard treatment [50].

After intravenous administration, (^131^I)-MIBG rapidly distributes to tumor cells and organs. Distribution studies showed uptake in liver (33%), lungs (0.8%), heart (0.8%), and salivary glands (0.4%) [13,52]. This may be attributed to sympathetic innervation and high vascularity of these organs [53]. MIBG is a small molecule that is insoluble in water and all organic solvents [13]. Only a small amount remains within the vascular compartment, were it accumulates in thrombocytes [52]. The volume of distribution is 2.9 mL/kg [13]. Uptake of MIBG into tumor cells is associated with tumor volume [54]. (^131^I)-MIBG is excreted, mainly unchanged, via glomerular filtration (70–90%) with a terminal half-life of radioactivity of 9–130 h [13,55].

No literature is available on (^131^I)-MIBG in obese patients, however, there were several studies in which (^123^I)-MIBG was administered to obese patients for cardiac scintigraphy. In these studies, patients received a diagnostic dose of 111 MBq (^123^I)-MIBG. All showed a lower cardiac uptake in obese patients due to reduced adrenergic innervation [56,57,58]. Unfortunately, these studies did not describe MIBG pharmacokinetics in obese patients. There are many studies in which (^131^I)-MIBG treatment was investigated in different tumor types and dosages. In most, a single dose of 3.7–7.4 GBq was administered. Generally, treatment with (^131^I)-MIBG is tolerated well with mild transient hematotoxicity being reported [13]. Response rates are around 30% [59]. Clinical studies did not include obese patients or did not report body weight in the final manuscripts.

Taking all data into account, there is insufficient evidence for a dose recommendation of (^131^I)-MIBG in obese patients. The distribution profile suggests that MIBG does not accumulate in fat tissue, however this was not investigated. Based on the distribution profile of (^131^I)-MIBG and its minimal toxicity profile, a fixed dose seems appropriate for treatment of patients with obesity.

### 5.2. Lutetium-177-Dotatate

(^177^Lu)-dotatate is approved for treatment of patients with unresectable or metastatic somatostatin receptor-positive GEP-NETs in a dose of 7.4 GBq every 8 weeks for a total of four doses [12]. (^177^Lu)-dotatate binds to subtype 2 somatostatin receptor (sst2) with high affinity. This somatostatin receptor is highly expressed in the majority of differentiated NETs and, therefore, (^177^Lu)-dotatate may be used to target this tumor type [60]. (^177^Lu)-dotatate emits beta (497 keV, 384 keV, and 176 keV) and gamma (208 keV and 113 keV) radiation with a half-life of 6.7 days [61].

In a dose-finding study, the (^177^Lu)-dotatate cumulative doses ranged from 27.8 to 29.6 GBq (generally administered in four cycles with 6–10 week treatment intervals). The maximum tolerated dose was not reached due to a mild safety profile [62]. In the following phase 3 trial, patients received the highest dose administered during phase 1/2 being 7.4 GBq every 8 weeks with a total of four cycles (cumulative dose 29.6 GBq) [63].

After intravenous administration, (^177^Lu)-dotatate rapidly distributes to the kidney, tumor tissue, liver, and spleen [64]. The protein binding of non-radioactive (^175^Lu)-dotatate is 43% and the volume of distribution is 460 L at 4 h post-infusion. Administration of 7.4 GBq resulted in an AUC of 41 ng*h/mL with a Cmax of 10 ng/mL [63]. (^177^Lu)-dotatate does not undergo hepatic metabolism, but is primarily cleared via the kidneys with 65% of drug being recovered in urine within 48 h after administration [63].

No literature is available on (^177^Lu)-dotatate treatment in obese patients. According to the FDA, no alternative dosing regimen is needed for subpopulations including patients with obesity, because no correlation was found between dose-normalized long-term hematologic and renal toxicity and body weight or BSA. It is unclear which data were used for these correlation analyses. In the ERASMUS trial, a large single-center phase 1/2 trial, patients with somatostatin-receptor-positive GEP-NET tumors were treated with 7.4 GBq (^177^Lu)-dotatate. The median BMI of included patients ranged from 15 to 45 kg/m^2^ with a median of 24 kg/m^2^. Although no body-weight-associated subgroup analyses were described, it shows that obese patients were included in (^177^Lu)-dotatate clinical trials and treated with a fixed dose of 7.4 GBq [61].

Based on the limited data available there is insufficient evidence for dose recommendations of (^177^Lu)-dotatate in obese patients. The FDA states that alternative dosing is not needed in obesity. Furthermore, obese patients were included in clinical trials and biodistribution studies suggest that (^177^Lu)-dotatate does not accumulate in fat tissue. Based on the limited clinical data, the distribution profile of (^177^Lu)-dotatate and its minimal toxicity profile, a fixed dose is recommended for treatment of patients with obesity.

### 5.3. Lutetium-177-PSMA

(^177^Lu)-PSMA is a promising novel treatment for patients with metastatic castration-resistant prostate cancer [64]. PSMA is a prostate-specific transmembrane glycoprotein, which is upregulated in 90–100% of prostate cancers [15]. Two types of (^177^Lu)-PSMA molecules were used in clinical trials, being (^177^Lu)-PSMA-617 and (^177^Lu)-PSMA-I&T. Although these molecules are built with other chelator agents, biodistribution was comparable [65]. ^177^Lu emits beta particles (497 keV, 384 keV, and 176 keV) and gamma photons (208 keV and 113 keV) with a half-life of 6.7 days [5].

The optimal administered activity is still under investigation. Doses ranged from 3.7 to 9.3 GBq in clinical studies [5]. There is one dose-escalation study available in which (^177^Lu)-PSMA-617 dosages of 4, 6, 7.4, and 9.3 GBq were administered to ten patients each [66]. Partial remission occurred in seven out of ten patients receiving 9.3 GBq compared to two out of ten in patients receiving 4 GBq. The toxicity profile was mild, with dose-independent acute hematologic toxicity (grade 3/4) in only two patients. However, the platelet count was decreased to 204.7/nL in the highest treatment group. A recently published phase 3 trial, established treatment efficacy of (^177^Lu)-PSMA-617 with a dose of 7.4 GBq every 6 weeks for four to six cycles [49]. A review and meta-analysis in which 13 clinical studies were included confirmed the low toxicity profile of (^177^Lu)-PSMA-617/I&T [67].

(^177^Lu)-PSMA accumulates in tissue with high expression of PSMA. In addition to tumor tissue, PSMA is expressed in the small intestine, proximal renal tubules, and salivary glands [65,68]. Pretherapeutic doses of (^177^Lu)-PSMA-617 showed the highest organ-absorbed dose in the kidney, salivary glands, liver, and bone using positron emission tomography/computed tomography (PET/CT) imaging [69]. Based on this biodistribution, the kidney, salivary glands, and bone marrow are considered dose-limiting organs [65]. (^177^Lu)-PSMA-167/I&T is cleared rapidly via urinary excretion [64]. In a simulation study, the effect of tumor volume in the effective dose of (^177^Lu)-PSMA-I&T was investigated [70]. The authors concluded that patients with large PSMA-positive tumor volumes might benefit from higher activities. These data suggest that individualized dosimetry could maximize treatment efficacy.

No information is available on dosing of (^177^Lu)-PSMA in patients with obesity. From PET tracer studies it is known that adipose tissue minimally accumulates PSMA radiotracer, but contributes to total body weight [71]. Based on the biodistribution profile, including limited uptake in adipose tissue, and the mild toxicity profile of (^177^Lu)-PSMA, a fixed dose seems appropriate for treatment of patients with obesity.

## 6. Discussion

In this review, data on the impact of obesity on the safety and efficacy of systemic radionuclide treatment are summarized. Available literature was evaluated and resulted in dose recommendations as presented in Table 2. Evidence was not equally strong for all therapeutic agents and so this review highlights the need for additional research on radiopharmaceuticals in obese patients. A limitation of this review article was the minimal clinical data on this topic. Even so, treating obese patients with radiopharmaceuticals is daily clinical practice and dosing guidelines are lacking. Therefore, with this review, we hope to have translated available literature into practical recommendations which may help guide clinicians in treating this patient population.

Different dosing strategies are used for radiopharmaceuticals. These can be classified into weight-based dosing, fixed dosing, and dosimetry-based dosing. Regarding weight-based dosing, the most commonly used body size descriptors are TBW, LBW, and FFM. LBW and FFM reflect the weight of non-fat body components, including organ and muscle function [72]. In contrast to FFM, LBW includes cellular membranes in calculating body weight [73]. As the proportion of cellular membranes to lean body mass is low (3–5%) these two descriptors may be used similarly [74]. It is known that LBW relates well to drug clearance [75]. Compared to normal-weight patients, patients with obesity have an excess of adipose tissue and an increased lean body mass. The ratio of LBW and adipose body weight is 4:1 in normal-weight patients compared to 3:2 in obese patients [72].

The majority of therapeutic radiopharmaceuticals are used for treatment of various cancer types. Literature suggests that obesity is associated with an increased risk of malignancies [76]. There is convincing evidence associating excessive weight and an increased risk for thyroid cancer and limited evidence for the association between excessive weight and advanced-stage prostate cancer [76]. This emphasizes the need for practical dose recommendations of radiopharmaceuticals for treatment of various cancer types in patients with obesity. Dosing on TBW could potentially lead to increased toxicity in obese patients, whereas dose calculation using LBM could lead to under-treatment. ^223^Ra was the only drug that was studied in subgroups of patients including obese patients, showing increased efficacy in terms of prolonged survival when dosing on TBW, with additional but moderate toxicity. For (^135^Sm)-EDTMP no such data are available, but based on dose-escalation studies, doses up to 93 MBq/kg are considered safe and effective in terms of hematologic toxicity and overall survival. As this is three times the registered dose, dosing on TBW (37 MBq/kg) in obese patients could possibly lead to good efficacy with a safe toxicity profile. Based on these data, both agents may be dosed on TBW in obese patients with close monitoring of adverse reactions.

The majority of therapeutic radionuclides are administered in a fixed-dosing scheme. Fixed dosing of radiopharmaceuticals in obese patients may be an appropriate strategy for drugs that selectively accumulate in target tissue. Although there is a potential risk of underdosing for lipophilic drugs with high levels of distribution to adipose tissue, the drugs described in this review do not accumulate in fat tissue. Based on their limited biodistribution and high accumulation at the site of action, fixed dosing seems appropriate for drugs such as ^89^Sr, (^186^Re)-HEDP, ^131^I, (131I^131^I MIBG, (^177^Lu)-dotatate, and (^177^Lu)-PSMA. Still, most radiopharmaceuticals show organ-specific toxicity, mostly related to exposure to radioactivity, such as hematologic toxicity. Therefore, close monitoring of toxicity is essential. Altogether, lack of clinical data on the pharmacokinetic distribution of therapeutic radiopharmaceuticals in obese patients may have relevant clinical implications.

A potential treatment method to overcome dosing uncertainty in obese patients is dosimetric methodology, in which the therapeutic dose is calculated based on target volume and an estimation of the absorbed radiation dose after administration of the radiopharmaceutical [77,78]. Dosimetry could help treatment response and limit toxicity by personalized dosing of radiopharmaceuticals. However, in clinical practice dosimetry-based dosing is not regularly used as registered doses are fixed or weight-based. For obese patients, dosing based on dosimetry may improve efficacy and decrease toxicity by personalized dosing.

In summary, limited data are available on dosing therapeutic radiopharmaceuticals in obese patients. Based on this literature review, we see clear opportunities to improve and optimize radiopharmaceutical treatment in obese patients.

## 7. Conclusions

This review summarizes relevant literature on radiopharmaceutical dosing and pharmacokinetic properties and aims to translate these data into practical guidelines for dosing of radiopharmaceuticals in obese patients. For ^223^Ra there is acceptable evidence that the registered dose of 55 kBq/kg may also be suitable for obese patients. Furthermore, for ^135^Sm-EDTMP, dose-escalation studies show that the maximum tolerated dose will probably not be reached in obese patients when dosing on MBq/kg. For ^89^Sr, (^186^Re)-HEDP, ^131^I, (^131^I)-MIBG, (^177^Lu)-dotatate, and (^177^Lu)-PSMA, there is insufficient evidence to support specific dose recommendations for heavyweight patients. From a pharmacokinetic view, fixed dosing may be appropriate. More research in obese patient populations is needed, especially in the light of increasing prevalence of obesity worldwide. Data presented in this review accentuate opportunities for future studies and for optimization of treatment with therapeutic radiopharmaceuticals in patients with obesity.

## Figures and Tables

**Table 1:** Overview of target organs, treatment applications, and mechanisms of action of radiopharmaceuticals included in this review article.

Radiotherapeutic Drug	Target	Treatment Application	Mechanism of Action
Radium-223	Bone tissue	Bone metastases	Mimics calcium and accumulates in bone matrix with high osteoblastic activity
Strontium-89			
Samarium-153-EDTMP	Bone tissue	Bone metastases	Mimics phosphate and accumulates in bone matrix with high bone turnover
Rhenium-186-HEDP			
Sodium iodide-131	Thyroid tissue	Hyperthyroidism (benign/malign)	Mimics iodide and accumulates in thyroid tissue
Iodide 131-MIBG	Norepinephrine transporter	Neuroendocrine tumors (NETs)	Structurally related to norepinephrine and binds to tumor tissue with high expression of the norepinephrine transporter
Lutetium-177-dotatate	Somatostatin receptor	Gastroenteropancreatic neuroendocrine tumors (GEP-NETs)	Structurally related to norepinephrine and binds to tumor tissue with high expression of the somatostatin receptors (subtype 2)
Lutetium-177-PSMA	PSMA	Metastatic castration-resistant prostate cancer	Structurally related to PSMA ligands and binds to tumor cells with high expression of the PSMA transmembrane protein.

EDTMP, ethylenediaminetetramethylene; HEDP, hydroxyethylidene diphosphonate; MIBG, metaiodobenzylguanidine; PSMA, prostate-specific membrane antigen.

**Table 2:** Overview of dosing regimens of radiopharmaceuticals and recommendations for dosing in obese patients.

Radiotherapeutic Drug	Dose in Non-Obese	Dose Regimen	Recommended Dose in Obese	Level of Evidence	References
Radium-223	55 kBq/kg TBW	Weight-based	Not different in obese	1	[7,24]
Samarium-153-EDTMP	37 MBq/kg TBW	Weight-based	Not different in obese	3	[25,26,29]
Strontium-89 *	150 MBq 1.5 MBq/kg TBW	Fixed dose	150 MBq 2 MBq/kg LBW	3	[8,32,33,34,35,36,37]
Rhenium-186-HEDP	1.110–1.295 GBq	Fixed dose	Not different in obese	5	[14,39,40]
Sodium iodide-131	Individual dose based on thyroid gland size ^π^	Fixed dose	Not different in obese	5	[10,16,43,46]
Iodide 131-MIBG	3.7–7.4 GBq	Fixed dose	Not different in obese	5	[13,52]
Lutetium-177-dotatate	7.4 GBq	Fixed dose	Not different in obese	3	[12,61,62,63,64]
Lutetium-177-PSMA	7.4 GBq	Fixed dose	Not different in obese	5	[49,65,67,68,71]

EDTMP, ethylenediaminetetramethylene; HEDP, hydroxyethylidene diphosphonate; LBW, lean body weight; MIBG, metaiodobenzylguanidine; PSMA, prostate-specific membrane antigen; TBW, total body weight. * TBW for non-obese patients, and LBW only in patients >75 kg otherwise fixed dose of 150 MBq. ^π^ There is no maximum defined dose for treatment of benign hyperthyroidism, while the maximum dose for malign application is 7.4 GBq per cycle without a maximum number of treatment cycles.

## References

[1] Flegal K.M., Kruszon-Moran D., Carroll M.D., Fryar C.D., Ogden C.L. (2016). Trends in Obesity Among Adults in the United States, 2005 to 2014. JAMA.

[2] World Health Organisation Obesity and Overweight. https://www.who.int/news-room/fact-sheets/detail/obesity-and-overweight#:~:text=In%202016%2C%2039%25%20of%20adults,tripled%20between%201975%20and%202016.

[3] Cho S.J., Yoon I.S., Kim D.D. (2013). Obesity-Related Physiological Changes and Their Pharmacokinetic Consequences. J. Pharm. Investig..

[4] Smit C., de Hoogd S., Brüggemann R.J.M., Knibbe C.A.J. (2018). Obesity and Drug Pharmacology: A Review of the Influence of Obesity on Pharmacokinetic and Pharmacodynamic Parameters. Expert Opin. Drug Metab. Toxicol..

[5] Kratochwil C., Fendler W.P., Eiber M., Baum R., Bozkurt M.F., Czernin J., Delgado Bolton R.C., Ezziddin S., Forrer F., Hicks R.J. (2019). EANM Procedure Guidelines for Radionuclide Therapy with ^177^Lu-Labelled PSMA-Ligands (^177^Lu-PSMA-RLT). Eur. J. Nucl. Med. Mol. Imaging.

[6] Ghanem M.A., Kazim N.A., Elgazzar A.H. (2011). Impact of Obesity on Nuclear Medicine Imaging. J. Nucl. Med. Technol..

[7] Food and Drug Administration (2012). Xofigo (Radium-223) Clinical Pharmacology and Biopharmaceutics Review.

[8] Food and Drug Administration (2019). Metastron Summary of Product Characteristics.

[9] Morris M.J., Corey E., Guise T.A., Gulley J.L., Kevin Kelly W., Quinn D.I., Scholz A., Sgouros G. (2019). Radium-223 Mechanism of Action: Implications for Use in Treatment Combinations. Nat. Rev. Urol..

[10] European Medicines Agency Summary of Product Characteristics of Sodium Iodide (131I). https://www.ema.europa.eu/en/documents/scientific-guideline/guideline-core-smpc-package-leaflet-sodium-iodide-131i-therapeutic-use_en.pdf.

[11] European Medicines Agency (2007). Quadramet Summary of Product Characteristics.

[12] European Medicines Agency Summary of Product Characteristics Lutathera. https://www.ema.europa.eu/en/documents/product-information/lutathera-epar-product-information_en.pdf.

[13] European Medicines Agency (2016). Summary of Product Characteristics [131I] Meta-Iodobenzylguanidine.

[14] Maxon H.R., Thomas S.R., Hertzberg V.S., Schroder L.E., Englaro E.E., Samaratunga R., Scher H.I., Moulton J.S., Deutsch E.A., Deutsch K.F. (1992). Rhenium-186 Hydroxyethylidene Diphosphonate for the Treatment of Painful Osseous Metastases. Semin. Nucl. Med..

[15] Ghosh A., Heston W.D. (2004). Tumor Target Prostate Specific Membrane Antigen (PSMA) and Its Regulation in Prostate Cancer. J. Cell. Biochem..

[16] Al-Ammar Y., Al-Mansour B., Al-Rashood O., Tunio M.A., Islam T., Al-Asiri M., Al-Qahtani K.H. (2018). Impact of Body Mass Index on Survival Outcome in Patients with Differentiated Thyroid Cancer. Braz. J. Otorhinolaryngol..

[17] Burns P.B., Rohrich R.J., Chung K.C. (2011). The Levels of Evidence and Their Role in Evidence-Based Medicine. Plast. Reconstr. Surg..

[18] Choi J.Y. (2018). Treatment of Bone Metastasis with Bone-Targeting Radiopharmaceuticals. Nucl. Med. Mol. Imaging.

[19] Savvidis C., Tournis S., Dede A.D. (2018). Obesity and Bone Metabolism. Hormones.

[20] de Laet C., Kanis J.A., Oden A., Johanson H., Johnell O., Delmas P., Eisman J.A., Kroger H., Fujiwara S., Garnero P. (2005). Body Mass Index as a Predictor of Fracture Risk: A Meta-Analysis. Osteoporos. Int..

[21] Salamat M.R., Salamat A.H., Janghorbani M. (2016). Association between Obesity and Bone Mineral Density by Gender and Menopausal Status. Endocrinol. Metab..

[22] Kim C.J., Oh K.W., Rhee E.J., Kim K.H., Jo S.K., Jung C.H., Won J.C., Park C.Y., Lee W.Y., Park S.W. (2009). Relationship between Body Composition and Bone Mineral Density (BMD) in Perimenopausal Korean Women. Clin. Endocrinol..

[23] Parker C., Nilsson S., Heinrich D., Helle S.I., O’Sullivan J.M., Fosså S.D., Chodacki A., Wiechno P., Logue J., Seke M. (2013). Alpha Emitter Radium-223 and Survival in Metastatic Prostate Cancer. N. Engl. J. Med..

[24] Nilsson S., Larsen R.H., Fosså S.D., Balteskard L., Borch K.W., Westlin J.E., Salberg G., Bruland Ø.S. (2005). First Clinical Experience with α-Emitting Radium-223 in the Treatment of Skeletal Metastases. Clin. Cancer Res..

[25] Farhanghi M., Holmes R.A., Volkert W.A., Logan K.W., Singh A. (1992). Toxicity and Pain Response Using an Escalating Dose Schedule in Treatment of Metastatic Bone Cancer. J. Nucl. Med..

[26] Eary J.F., Collins C., Stabin M., Vernon C., Petersdorf S., Baker M., Hartnett S., Ferency S., Addison S.J., Appelbaum F. (1993). Samarium-153-EDTMP Biodistribution and Dosimetry Estimation. J. Nucl. Med..

[27] Bayouth J.E., Macey D.J., Kasi L.P., Fossella F. (1994). Dosimetry and Toxicity of Samarium-153-EDTMP Administered for Bone Pain Due to Skeletal Metastases. J. Nucl. Med..

[28] Singh A., Holmes R.A., Farhangi M., Volkert W.A., Williams A., Stringham L.M., Ketring A.R. (1989). Human Pharmacokinetics of Samarium-153 EDTMP in Metastatic Cancer. J. Nucl. Med..

[29] Collins C., Eary J.F., Donaldson G., Vernon C., Bush N.E., Petersdorf S., Livingston R.B., Gordon E.E., Chapman C.R., Appelbaum F.R. (1993). Samarium-153-EDTMP in Bone Metastases of Hormone Refractory Prostate Carcinoma: A Phase I/II Trial. J. Nucl. Med..

[30] Dickinson C.Z., Hendrix N.S. (1993). Strontium-89 Therapy in Painful Bony Metastases. J. Nucl. Med. Technol..

[31] Therapeutic Goods Administration Australia Preclinical Evaluation of Application for Registration: Metastron. https://www.tga.gov.au/sites/default/files/foi-180-1213-3.pdf.

[32] Robinson R.G., Blake G.M., Preston D.F., McEwan A.J., Spicer J.A., Martin N.L., Wegst A.V., Ackery D.M. (1989). Strontium-89: Treatment Results and Kinetics in Patients with Painful Metastatic Prostate and Breast Cancer in Bone. Radiographics.

[33] Silberstein E., Taylor A. (1996). Procedure Guideline for Bone Pain Treatment: 1.0. J. Nucl. Med..

[34] Tennvall J., Darte L., Lundgren R., Mohamed El Hassan A. (1988). Palliation of Multiple Bone Metastases from Prostatic Carcinoma with Strontium-89. Acta Oncol..

[35] Kloiber R., Molnar C.P., Barnes M. (1987). Sr-89 Therapy for Metastatic Bone Disease: Scintigraphic and Radiographic Follow-Up. Radiology.

[36] Laing A.H., Ackery D.M., Bayly R.J., Buchanan R.B., Lewington V.J., McEwan A.J.B., Macleod P.M., Zivanovic M.A. (1991). Strontium-89 Chloride for Pain Palliation in Prostatic Skeletal Malignancy. Br. J. Radiol..

[37] Robinson R.G., Preston D.F., Spicer J.A., Baxter K.G. (1992). Radionuclide Therapy of Intractable Bone Pain: Emphasis on Strontium-89. Semin. Nucl. Med..

[38] Klett R., Lange U., Haas H., Voth M., Pinkert J. (2007). Radiosynoviorthesis of Medium-Sized Joints with Rhenium-186-Sulphide Colloid: A Review of the Literature. Rheumatology.

[39] Maxon H.R., Deutsch E.A., Thomas S.R., Libson K., Lukes S.J., Williams C.C., Ali S. (1988). Re-186(Sn) HEDP for Treatment of Multiple Metastatic Foci in Bone: Human Biodistribution and Dosimetric Studies. Radiology.

[40] de Klerk J.M., van Dijk A., van het Schip A.D., Zonnenberg B.A., van Rijk P.P. (1992). Pharmacokinetics of Rhenium-186 after Administration of Rhenium-186-HEDP to Patients with Bone Metastases. J. Nucl. Med..

[41] Maxon H.R., Schroder L.E., Thomas S.R., Hertzberg V.S., Deutsch E.A., Scher H.I., Samaratunga R.C., Libson K.F., Williams C.C., Moulton J.S. (1990). Re-186(Sn) HEDP for Treatment of Painful Osseous Metastases: Initial Clinical Experience in 20 Patients with Hormone-Resistant Prostate Cancer. Radiology.

[42] Maxon H.R., Schroder L.E., Hertzberg V.S., Thomas S.R., Englaro E.E., Samaratunga R., Smith H., Moulton J.S., Williams C.C., Ehrhardt G.J. (1991). Rhenium-186(Sn)HEDP for Treatment of Painful Osseous Metastases: Results of a Double-Blind Crossover Comparison with Placebo. J. Nucl. Med..

[43] Luster M., Clarke S.E., Dietlein M., Lassmann M., Lind P., Oyen W.J.G., Tennvall J., Bombardieri E. (2008). Guidelines for Radioiodine Therapy of Differentiated Thyroid Cancer. Eur. J. Nucl. Med. Mol. Imaging.

[44] Drent M.L., van der Veen E.A. (1995). Endocrine Aspects of Obesity. Neth. J. Med..

[45] Verburg F.A., de Keizer B., Isselt J.W. (2007). Use of Radiopharmaceuticals for Diagnosis, Treatment, and Follow-Up of Differentiated Thyroid Carcinoma. Anti-Cancer Agents Med. Chem..

[46] Food and Drug Administration Drug Label of Sodium Iodide (131I). https://www.accessdata.fda.gov/drugsatfda_docs/label/2011/021305s025lbl.pdf.

[47] Itani M., Lewis D.H. (2017). I-131 Uptake in Fat Necrosis of the Breast. Radiol. Case Rep..

[48] Iwano S., Ito S., Kamiya S., Ito R., Kato K., Naganawa S. (2020). Unexpected Radioactive Iodine Accumulation on Whole-Body Scan after I-131 Ablation Therapy for Differentiated Thyroid Cancer. J. Med. Sci..

[49] Sartor O., de Bono J., Chi K.N., Fizazi K., Herrmann K., Rahbar K., Tagawa S.T., Nordquist L.T., Vaishampayan N., El-Haddad G. (2021). Lutetium-177-PSMA-617 for Metastatic Castration-Resistant Prostate Cancer. N. Engl. J. Med..

[50] Kayano D., Kinuya S. (2018). Current Consensus on I-131 MIBG Therapy. Nucl. Med. Mol. Imaging.

[51] Giammarile F., Chiti A., Lassmann M., Brans B., Flux G. (2008). EANM Procedure Guidelines for ^131^I-Meta-Iodobenzylguanidine (^131^I-MIBG) Therapy. Eur. J. Nucl. Med. Mol. Imaging.

[52] Wafelman A.R., Hoefnagel C.A., Maes R.A.A., Beijnen J.H. (1994). Radioiodinated Metaiodobenzylguanidine: A Review of Its Biodistribution and Pharmacokinetics, Drug Interactions, Cytotoxicity and Dosimetry. Eur. J. Nucl. Med..

[53] Nakajo M., Shapiro M., Copp J., Kalff V., Gross M.D., Sisson J.C., Belerwaltes W.H. (1983). The Normal and Abnormal Distributionof the Adrenomeduuary Imaging Agent M-[I-131]IodobenzyIguanid (I-131MIBG) in Man: Evaluationby Scintigraphy. J. Nucl. Med..

[54] Bomanji J., Levison D.A., Flatman W.D., Horne T., Boulous P.M.-G., Ross G., Britton K.E., Besser G.M. (1987). Uptake of Ioine-123 MIBG by Pheochromocytomas, Paragangliomas, and Neuroblastomas: A Histopathological Comparison. J. Nucl. Med..

[55] Lashford L.S., Moyes J., Ott R., Fielding S., Babich J., Mellors S., Gordon I., Evans K., Kemshead J.T. (1988). The Biodistribution and Pharmacokinetics of Meta-Iodobenzylguanidine in Childhood Neuroblastoma. Eur. J. Nucl. Med..

[56] Sunaga A., Hikoso S., Yamada T., Yasumura Y., Uematsu M., Abe H., Nakagawa Y., Higuchi Y., Fuji H., Mano T. (2021). Abdominal Obesity, and Not General Obesity, Is Associated with a Lower 123I MIBG Heart-to-Mediastinum Ratio in Heart Failure Patients with Preserved Ejection Fraction. Eur. J. Nucl. Med. Mol. Imaging.

[57] Komici K., Bencivenga L., Paolillo S., Gargiulo P., Formisano R., Assante R., Nappi C., Marsico F., D’Antonio A., de Simini G. (2019). Impact of Body Mass Index on Cardiac Adrenergic Derangement in Heart Failure Patients: A 123I-MIBG Imaging Study. Eur. J. Nucl. Med. Mol. Imaging.

[58] Pellegrino T., Piscopo V., Boemio A., Russo B., de Matteis G., Pellegrino S., Giorgio S.M., Amato M., Petretta M., Cuocolo A. (2015). Impact of Obesity and Acquisition Protocol on (123)I-Metaiodobenzylguanidine Indexes of Cardiac Sympathetic Innervation. Quant. Imaging Med. Surg..

[59] Loh K.C., Fitzgerald P.A., Matthay K.K., Yeo P.P.B., Price D.C. (1997). The Treatment of Malignant Pheochromocytoma with Lodine-131 Metaiodobenzylguanidine (^131^1-MIBG): A Comprehensive Review of 116 Reported Patients. J. Endocrinol. Investig..

[60] Krenning E.P., Kwekkeboom D.J., Bakker W.H., Breeman W.A.P., Kooij P.P.M., Oei H.Y., van Hagen M., Postema P.T.E., de Jong M., Reubi J.C. (1993). Somatostatin Receptor Scintigraphy with [^111^1n-DTPA-D-Phe^l^] and [^123^1-Tyr^3^]-Octreotide: The Rotterdam Experience with More than 1000 Patients. Eur. J. Nucl. Med..

[61] Food and Drug Administration Multidisciplinary Review and Evaluation Lutathera. https://www.accessdata.fda.gov/drugsatfda_docs/nda/2018/208700orig1s000multidiscipliner.pdf.

[62] Kwekkeboom D.J., de Herder W.W., Kam B.L., van Eijck C.H., van Essen M., Kooij P.P., Feelders R.A., van Aken M.O., Krenning E.P. (2008). Treatment with the Radiolabeled Somatostatin Analog [^177^Lu-DOTA 0,Tyr^3^]Octreotate: Toxicity, Efficacy, and Survival. J. Clin. Oncol..

[63] Strosberg J., El-Haddad G., Wolin E., Hendifar A., Yao J., Chasen B., Mittra E., Kunz P.L., Kulke M.H., Jacene H. (2017). Phase 3 Trial of ^177^Lu-Dotatate for Midgut Neuroendocrine Tumors. N. Engl. J. Med..

[64] van Kalmthout L.W.M., van der Sar E.C.A., Braat A.J.A.T., de Keizer B., Lam M.G.E.H. (2020). Lutetium-177-PSMA Therapy for Prostate Cancer Patients—a Brief Overview of the Literature. Tijdschr. Voor Urol..

[65] Okamoto S., Thieme A., Allmann J., D’Alessandria C., Maurer T., Retz M., Tauber R., Heck M.M., Wester H.J., Tamaki N. (2017). Radiation Dosimetry for ^177^Lu-PSMA I&T in Metastatic Castration-Resistant Prostate Cancer: Absorbed Dose in Normal Organs and Tumor Lesions. J. Nucl. Med..

[66] Rathke H., Giesel F.L., Flechsig P., Kopka K., Mier W., Hohenfellner M., Haberkorn U., Kratochwil C. (2018). Repeated ^177^Lu-Labeled PSMA-617 Radioligand Therapy Using Treatment Activities of Up to 9.3 GBq. J. Nucl. Med..

[67] Yadav M.P., Ballal S., Sahoo R.K., Dwivedi S.N., Bal C. (2019). Radioligand Therapy With ^177^Lu-PSMA for Metastatic Castration-Resistant Prostate Cancer: A Systematic Review and Meta-Analysis. AJR Am. J. Roentgenol..

[68] Kabasakal L., AbuQbeitah M., Aygun A., Yeyin N., Ocak M., Demirci E., Toklu T. (2015). Pre-Therapeutic Dosimetry of Normal Organs and Tissues of ^177^Lu-PSMA-617 Prostate-Specific Membrane Antigen (PSMA) Inhibitor in Patients with Castration-Resistant Prostate Cancer. Eur. J. Nucl. Med. Mol. Imaging.

[69] Khawar A., Eppard E., Sinnes J.P., Roesch F., Ahmadzadehfar H., Kurpig S., Meisenheimer M., Gaertner F.C., Essler M., Bundschuh R.A. (2018). Prediction of Normal Organ Absorbed Doses for [^177^Lu]Lu-PSMA-617 Using [^44^Sc]Sc-PSMA-617 Pharmacokinetics in Patients With Metastatic Castration Resistant Prostate Carcinoma. Clin. Nucl. Med..

[70] Begum N.J., Thieme A., Eberhardt N., Tauber R., D’Alessandria C., Beer A.J., Glatting G., Eiber M., Kletting P. (2018). The Effect of Total Tumor Volume on the Biologically Effective Dose to Tumor and Kidneys for ^177^Lu-Labeled PSMA Peptides. J. Nucl. Med..

[71] Zhao J., Xue Q., Chen X., You Z., Wang Z., Yuan J., Liu H., Hu L. (2021). Evaluation of SUVlean Consistency in FDG and PSMA PET/MR with Dixon-, James-, and Janma-Based Lean Body Mass Correction. EJNMMI Phys..

[72] Barras M., Legg A. (2017). Drug Dosing in Obese Adults. Aust. Prescr..

[73] Yu S., Visvanathan T., Field J., Ward L.C., Chapman I., Adams R., Wittert G., Visvanathan R. (2013). Lean Body Mass: The Development and Validation of Prediction Equations in Healthy Adults. BMC Pharmacol. Toxicol..

[74] Janmahasatian S., Duffull S.B., Chagnac A., Kirkpatrick C.M.J., Green B. (2008). Lean Body Mass Normalizes the Effect of Obesity on Renal Function. Br. J. Clin. Pharmacol..

[75] Morrish G.A., Pai M.P., Green B. (2011). The Effects of Obesity on Drug Pharmacokinetics in Humans. Expert Opin. Drug Metab. Toxicol..

[76] Avgerinos K.I., Spyrou N., Mantzoros C.S., Dalamaga M. (2019). Obesity and Cancer Risk: Emerging Biological Mechanisms and Perspectives. Metab. Clin. Exp..

[77] Stokke C., Gabina P.M., Solny P., Cicone F., Sandstrom M., Gleisner K.S., Chiesa C., Spezi E., Paphiti M., Konijnenberg M. (2017). Dosimetry-Based Treatment Planning for Molecular Radiotherapy: A Summary of the 2017 Report from the Internal Dosimetry Task Force. EJNMMI Phys..

[78] Chiesa C., Sjogreen Gleisner K., Flux G., Gear J., Walrand S., Bacher K., Eberlein U., Visser E.P., Chouin N., Ljungberg M. (2017). The Conflict between Treatment Optimization and Registration of Radiopharmaceuticals with Fixed Activity Posology in Oncological Nuclear Medicine Therapy. Eur. J. Nucl. Med. Mol. Imaging.

